# Mesoporous Silica Matrix as a Tool for Minimizing Dipolar Interactions in NiFe_2_O_4_ and ZnFe_2_O_4_ Nanoparticles

**DOI:** 10.3390/nano7070151

**Published:** 2017-06-22

**Authors:** Maider Virumbrales, Regino Saez-Puche, María José Torralvo, Veronica Blanco-Gutierrez

**Affiliations:** Departamento de Química Inorgánica, Facultad de Ciencias Químicas, Universidad Complutense de Madrid, 28040 Madrid, Spain; maidervirumbrales@pdi.ucm.es (M.V.); rsp92@quim.ucm.es (R.S.-P.); torralvo@quim.ucm.es (M.J.T.)

**Keywords:** superparamagnetic, ferrite, MCM-matrix, dipolar interactions, matrix effect

## Abstract

NiFe_2_O_4_ and ZnFe_2_O_4_ nanoparticles have been prepared encased in the MCM (Mobile Composition of Matter) type matrix. Their magnetic behavior has been studied and compared with that corresponding to particles of the same composition and of a similar size (prepared and embedded in amorphous silica or as bare particles). This study has allowed elucidation of the role exerted by the matrix and interparticle interactions in the magnetic behavior of each ferrite system. Thus, very different superparamagnetic behavior has been found in ferrite particles of similar size depending on the surrounding media. Also, the obtained results clearly provide evidence of the vastly different magnetic behavior for each ferrite system.

## 1. Introduction

Finite size effects are one of the most studied issues in current materials science, since they involve a modification of the materials properties (as has been extensively reported) [1,2,3,4]. Phenomenology (occurring on the surface of the particles) becomes more important when decreasing the particle size, and is responsible most of the time for this property modification. Thus, finite size effects are the consequence of several factors, such as the low coordination sphere characteristic of surface atoms [5] and different atoms’ distances in comparison with bulk materials [6]. When it comes to magnetic materials, if they are prepared below a critical particle size they are single-domain, since the magnetostatic energy cannot be compensated by the energy wall formation [7,8]. A larger magnetic moment is then obtained with the increment of the particle size in the single-domain region, as there are more coupled-moment carriers [9,10,11]. For a certain particle size and temperature range (between the blocking and ordering temperatures *T_B_* and *T_O_*, respectively), magnetic materials behave as superparamagnetic, so when a magnetic field is applied, the particles’ moments align in the field direction, resulting in high magnetization values with an absence of coercivity [7,8,12]. Superparamagnetism is deeply studied in the case of ferrite nanoparticles, since interesting properties can be found in these materials [13,14,15,16]. When reducing the particle size of MFe_2_O_4_ (M: Fe, Co, Ni, Zn, …) materials, there is a softening of the lattice vibration [6], leading to a cation distribution characteristic of the mixed spinel structure $\left(M_{1-x}Fe_{x}\right)\left[Fe_{2-x}M_{x}\right]O_{4}$, where *x* is the inversion degree (0 < *x* < 1) [17,18,19]. While bulk ZnFe_2_O_4_ behaves as antiferromagnetic when it is prepared in the micrometric range [20], it presents a ferrimagnetic ordering as consequence of a different cation distribution in the nanoscale [10]. In the same way, NiFe_2_O_4_ nanoparticles present mixed spinel structure leading to an internal ferrimagnetic ordering different to that illustrated by microparticles of same composition [18]. The net magnetization of the ferrite sample is directly related with the internal magnetic ordering and particle size, and is influenced by external factors such as interparticle interactions [21], which fall into two types: those occurring between surface spins of different particles at low temperatures that tends to minimize the magnetic response [22]; and those taking place at higher temperature values (between dipolar moments of different particles) that increase the magnetic response of the sample [18,23]. These external factors are of capital importance when considering these compounds as potential candidates to be employed with biomedical applications (such as drug-delivery agents), or whether they should be used for hyperthermia or as ferrofluids [24,25,26,27]. A superparamagnetic behavior dependent on temperature and the applied magnetic field has also been recently studied [19,28]. The surrounding media seems to also be very important as it may exert two important roles in the particles: (1) it can promote or minimize the interparticle interactions, which tends to minimize or favor the magnetic response; and (2) it may modify the surface anisotropy, leading to a harder or softer magnetic material [10,29]. In this sense, a magnetic hardening of ferrite particles at low temperatures has been observed when they are embedded in amorphous silica (due to the mechanical stress imposed by the matrix) [10,30], and zinc ferrite nanoparticles present a free particle moment rotation when they are contained in porous structures [31]. In addition, ZnFe_2_O_4_ and NiFe_2_O_4_ seem to be very different magnetic systems; while dipole interactions usually govern the magnetic behavior of nickel ferrite samples, these kind of interaction are not very important in zinc ferrite particles, so they almost preserve a non-perturbed magnetic behavior.

In this work we present the unexplored magnetic behavior of ZnFe_2_O_4_ and NiFe_2_O_4_ particles contained in the silica MCM-41 type matrix and compare their magnetic behavior with those particles of similar sizes, prepared as bare nanoparticles and embedded in amorphous silica (in order to have a better knowledge of the role of the different microstructure of the matrix in the magnetic behavior of the contained particles, since different interparticle interactions are expected).

## 2. Results and Discussion

NiFe_2_O_4_ and ZnFe_2_O_4_ particles with an average size ranging from 3 to 5 nm have been prepared as bare particles (Ni3 and Zn4 samples), and contained in the MCM-41 matrix (NiMH and ZnMH samples) and amorphous silica (NiM5 and ZnM3 samples) (note the brief description of them in Table 1). The full characterization of Ni3 and Zn4 samples can be found in [18,22], respectively, and [10,30] for NiM5 and ZnM3 samples, respectively.

**Figure 1:**
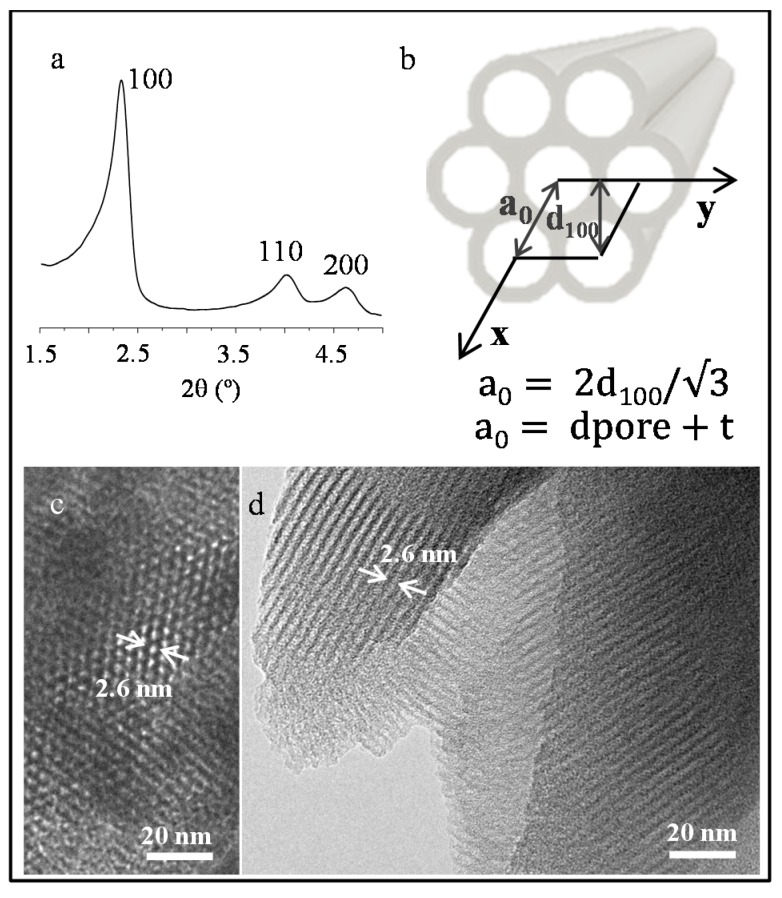
X-ray diffraction pattern corresponding to the MCM-41 type matrix (**a**); An illustrative picture of the matrix microstructure is shown in (**b**); Representative TEM images of the MCM-41 type matrix are also shown (**c**,**d**).

**Figure 2:**
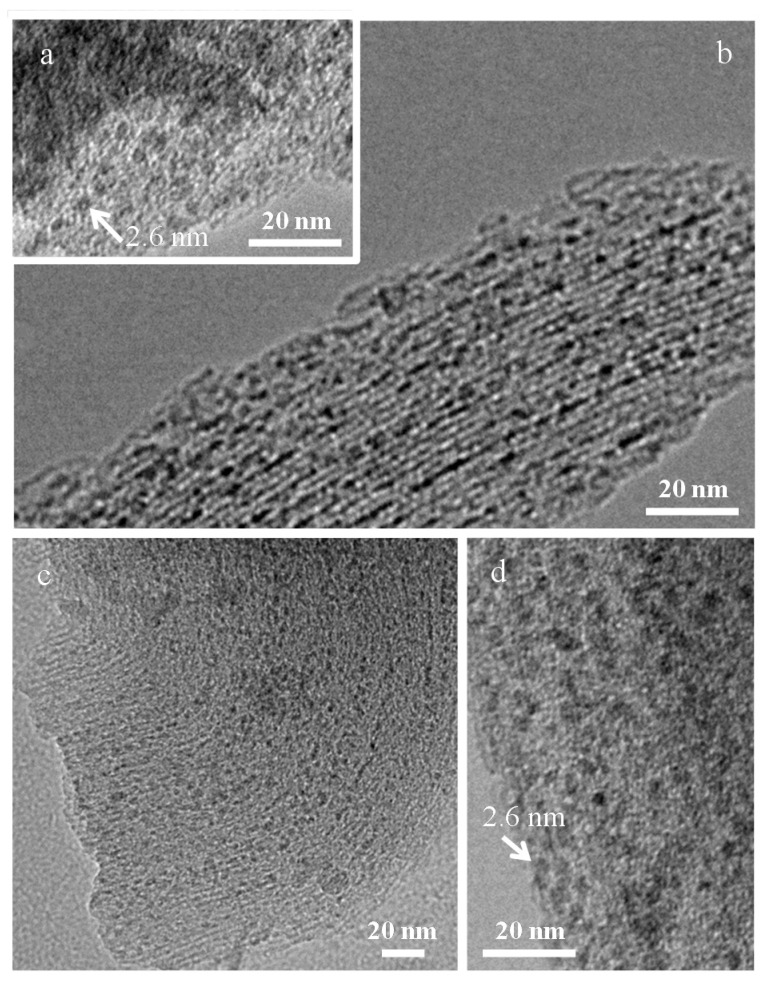
TEM images corresponding to Ni-MH (**a**,**b**) and Zn-MH (**c**,**d**) samples. Particles inside the channels are indicated by arrows in both samples.

The low-angle X-ray diffraction pattern of the silica mesoporous MCM matrix is shown in Figure 1a. The most intense maximum corresponds to the (100) reflection of a 2D hexagonal network. From the *d*_100_ spacing, the cell parameter *a*_0_ has been calculated using the expression:

$$a_{0}=2d_{100}/\sqrt{3}$$

obtaining a value of 4.3 nm. The size of the pore (2.6 nm) has been estimated from images obtained by transmission electron microscopy (TEM) (Figure 1c,d), and the thickness (*t*) of the silica framework (1.7 nm) has been calculated from the expression:

$$a_{0}=d_{pore}+t$$

taking into account the already estimated parameters (Figure 1b). Due to the small size of the encapsulated nanocrystals and the low ferrite:matrix ratio (discussed below) in the X-ray diffraction patterns of NiFe_2_O_4_ and ZnFe_2_O_4_, and particles encased in the MCM-type matrix (NiMH and ZnMH samples, respectively—not shown), only the reflections corresponding to the matrix can be visualized. This fact has already been observed by other authors in several cases [31,32]. Representative TEM images corresponding to NiMH and ZnMH samples are shown in Figure 2a,b,d and Figure 2c,d, respectively. Ferrite particles of approximately 2.6 nm can be distinguished along the channels (particles indicated by arrows in Figure 2) when the images are slightly out of focus.

The adsorption-desorption isotherms of the prepared MCM-type matrix and NiMH and ZnMH samples (Figure S1a of the Supporting Information) are type IV, with hysteresis loops extending from P/P_0_ ≈ 0.45 up to the high pressure region. For NiMH and ZnMH samples, the pores of the matrix are partially occupied by the particles, as can be elucidated from the quantity adsorbed at any relative pressure (which is higher in the case of the matrix than in the composites). The shape of the isotherms (characteristic of the MCM-type materials) [33] and the pore size distribution curves (Figure S1b) indicate that the porous structure of the matrix is preserved during the synthesis of the nanoparticles inside the channels. The Brunauer-Emmett-Teller (BET) equation [34] was used to calculate the surface area, while mesopore size distributions were calculated using the Barrett-Joyner-Halenda (BJH) method for a cylindrical pore model [35,36]. The BET area (S_BET_), cumulative pore volume (V_P_), and channel diameter (*d*_pore_) have been calculated for each sample from their corresponding isotherms and the obtained values are collected in Table S1 (Supporting Information). Nanoparticles contained in the pores create a new surface but also offer a partial blocking of the internal surface of the channels. As a result, the S_BET_ and V_P_ values for NiMH and ZnMH samples are lower than for the empty matrix. Also, the pore diameter values obtained from the adsorption measurement are consistent with the channel’s diameter (estimated from the TEM images).

The ferrite:matrix weight ratio has been estimated after energy-dispersive X-ray spectroscopy measurements obtaining average values of 15% and 20% (for the NiMH and ZnMH sample, respectively). The microstructure of NiM5, ZnM3 (5 nm NiFe_2_O_4_ and 3 nm ZnFe_2_O_4_ particles embedded in amorphous silica, respectively) and Ni3 and Zn4 (3 nm NiFe_2_O_4_ and 4 nm ZnFe_2_O_4_ bare particles) is fully described in previous works ([18,22] for the Ni3 and Zn4 samples, respectively, and [10,30] for the NiM5 and ZnM3 samples, respectively).

A magnetic study has been performed for these ferrite nanoparticles contained in the MCM matrix, and their behavior has been compared with that shown by particles of a similar size embedded in amorphous silica (NiM5 [30] and ZnM3 [10] samples) and prepared as bare nanoparticles (Ni3 [18] and Zn4 [22] samples). In different magnetic analyses the ferrite:matrix weight ratio has been taken into account in the case of NiMH, ZnMH, NiM5 and ZnM3 samples; therefore, the recorded data correspond to the ferrite system.

**Figure 3:**
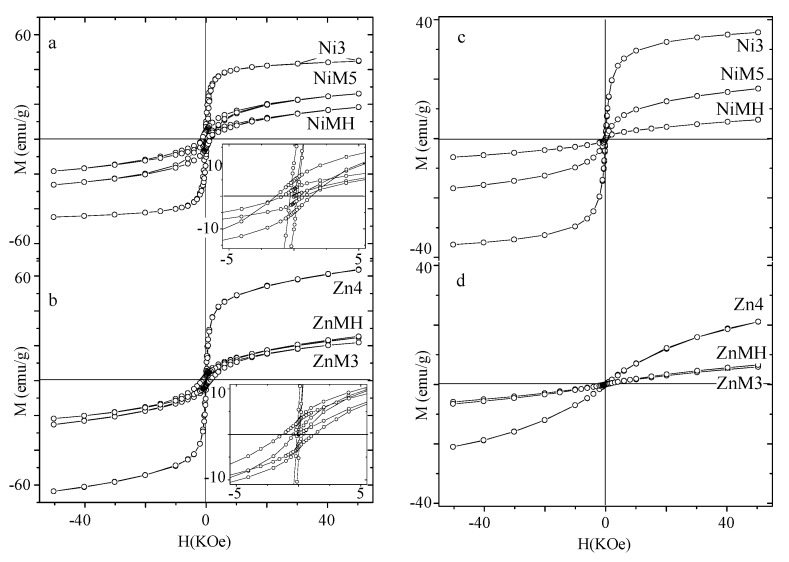
Magnetization curves measured at 5 K (Ni (**a**) and Zn (**b**) samples, respectively) and 250 K (Ni (**c**) and Zn (**d**) samples, respectively).

Magnetization (M) versus an applied magnetic field (H) has been measured at 5 K for all the samples. In Figure 3 the M(H) curves corresponding to particles of NiFe_2_O_4_ and ZnFe_2_O_4_ (Figure 3a,b, respectively) are depicted as bare particles, contained in the MCM-type matrix and embedded in amorphous silica.

The experimental data in the high applied magnetic field region have been fitted to the approach to saturation law for all the samples [28,37]:

(1) $$M(T)=M_{S}\left(1-\frac{a}{\sqrt{H}}-\frac{b}{H^{2}}\right)+cH\;b=\frac{8}{105}\frac{K^{2}}{{M_{S}}^{2}}$$

(where *a* and *b* are constants and *c* is the magnetic susceptibility at high magnetic fields). From the fitting, the saturation magnetization (*M_S_*) and anisotropy constant (K) have been obtained. Also, the anisotropy field (*H_K_*) has been calculated from the expression [38]:

(2) $$H_{K}=\frac{2K}{M_{S}}$$

The values have been collected in Table 1, together with the estimated coercive field (*H_C_*) at 5 K.

The saturation magnetization is determined by the number of coupled moments carriers of the particle, which is directly related to the inversion degree of the spinel structure and particle size [11,18]. Also, if there is a layer of disordered surface spins that do not contribute to the magnetization of the sample, the M_S_ value is lower than the expected one. M(H) curves measured at *T* < *T_B_* put into evidence the existence of this non-magnetic layer (composed by canted spins when a non-saturated magnetization is observed) [39]. Due to geometrical reasons in very small particles, surface spins may be forced to be canted, while a paramagnetic contribution would be observed in the M(H) curve. This non-magnetic surface layer may be thicker in the case of nanoparticles contained in matrices, thus showing a lower *M_S_* value [30,31]. Encased nickel ferrite particles present lower magnetization values more than bare particles and are a more important paramagnetic contribution (revealing a significant non-magnetic surface layer in these cases (Figure 3a)). This may justify the low magnetization values for the encased particles (as shown in ref. [30]), although the inversion degree should be taken into account for explaining the unexpected, slightly higher *M_S_* value of the Ni3 sample. A similar interpretation could be done in the case of zinc ferrite samples (the higher magnetization obtained for bare particles would be the result of an absence of an important non-magnetic surface layer, the particle size, and (probably) a higher inversion degree).

The effective anisotropy constant, which depends (among other factors) on the particle size [40] and interparticle interactions (dipolar interactions tend to decrease this value while surface spins interactions, happening at low temperature, tend to increase it) [22,41] seems to be affected by the matrix as well, which would increase the surface anisotropy resulting in a larger effective anisotropy constant [30]. Thus, it can be observed for both compositions that ferrite particles contained in the MCM-type matrix present the highest value of the effective anisotropy constant, probably as consequence of the increment of the surface anisotropy component as a matrix effect. This effect must be higher in particles contained in MCM-type matrix that in amorphous silica, probably due to a higher surface area of the ferrite particles in contact with the matrix (because of the smaller particle size and geometrical distribution along the channels).

Similarly, the anisotropy field *H_K_* indicates the intrinsic magnetic hardness of the material as it relates the total obtained magnetization of a sample to a certain anisotropy (see Equation (2)). This applies, therefore, when evaluating the magnetic hardness of the material (not only the anisotropy constant, but the total magnetization must be considered as well). Higher *H_K_* values are observed for those particles contained in the MCM-type matrix for both compositions, and the smallest values correspond to bare particles. The highly important mechanical stress imposed by the MCM-type matrix makes the encased ferrite particles behave as a very hard magnetic material.

The coercive field can be described by the following expression [38]:

(3) $$H_{C}=0.48H_{K}{\left[\left(1-\frac{T}{T_{B}}\right)\right]}^{a}$$

(where *a* is a parameter dependent on the interparticle interactions (0.5 < *a* < 1)). The estimated values for a 5 K measuring temperature are collected in the Table. The coercive field value is directly related to *H_K_*, and this dependency is affected by two factors: the blocking temperature (which tends to perturb more this dependency when it is smaller); and the particle interactions (which affect the parameter). The higher *T_B_* values, together with a different kind of interpaticle interactions influencing the a parameter, would explain the highest *H_C_* values observed for particles embedded in amorphous silica (although they present lower *H_K_* values in comparison with those exhibited by the ZnMH and NiMH samples).

M(H) curves measured at 250 K are shown in Figure 3c,d for the NiFe_2_O_4_ and ZnFe_2_O_4_ particles, respectively.

While the three NiFe_2_O_4_ samples illustrate an S-shaped curve characteristic of the superparamagnetic behavior, ZnFe_2_O_4_ particles exhibit M(H) curves with a large paramagnetic contribution for this temperature of measurement. A lower magnetization value is observed in ferrite particles contained or embedded in silica for both compositions, as it is also observed in M(H) curves measured at 5 K. This would indicate that for this temperature the internal ordering has not been destroyed in total for the Zn4 sample; otherwise, the M(H) curve would depict the same tendency for the three samples.

**Figure 4:**
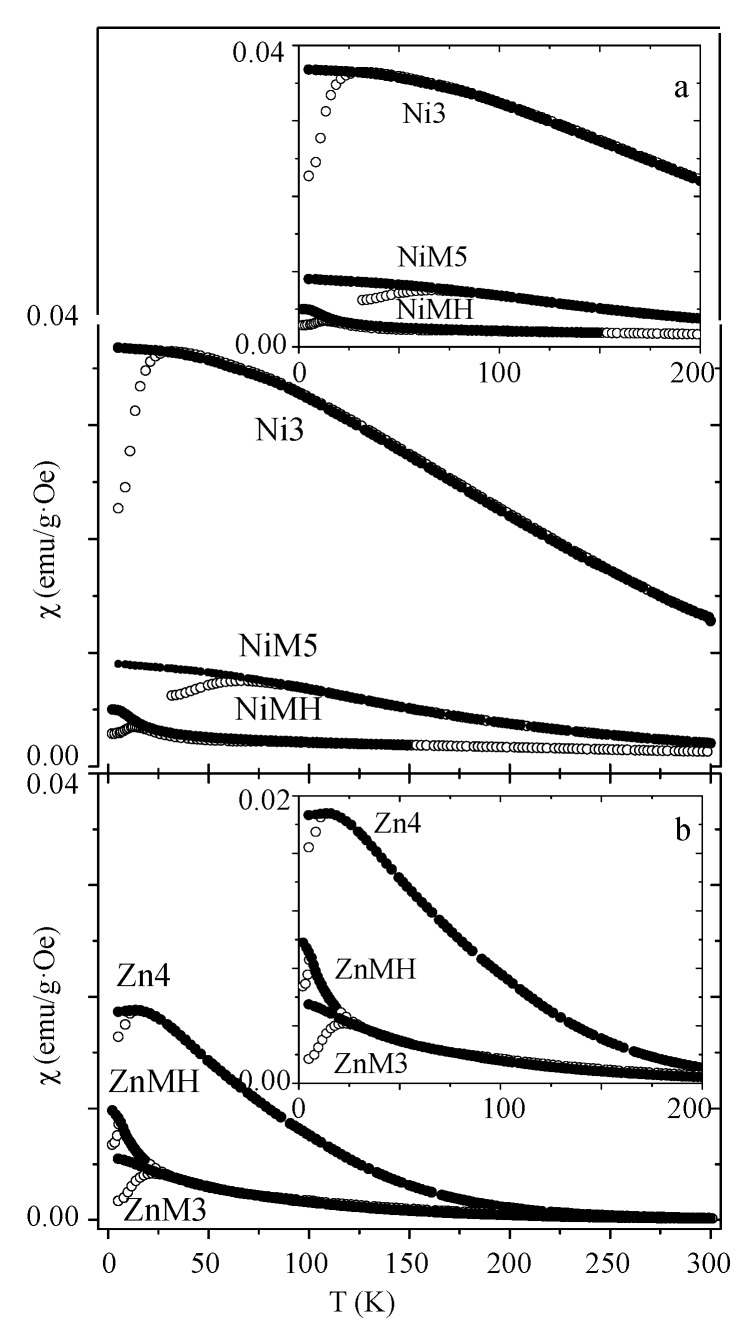
Magnetic susceptibility measured at 500 Oe, corresponding to nickel ferrite (**a**) and zinc ferrite (**b**) samples.

Magnetic susceptibility measured at 500 Oe is depicted in Figure 4 for both compositions.

The blocking temperature value, estimated from the maximum of the ZFC curve, is collected in Table 1 for all the samples. This parameter can be described by the following expression [8,30]:

(4) $$T_{B}=\frac{KV{\left(1-\frac{H}{H_{K}}\right)}^{2}}{25\kappa_{B}}$$

where *κ_B_* is the Boltzman constant.

This expression indicates that the blocking temperature is directly related with the anisotropy barrier *KV* (where *V* is the effective magnetic volume), which is slightly modified in the presence of an external field if the magnetic material is very hard (high *H_K_* value). On the contrary, in the case of soft magnetic materials (low *H_K_* value), the external magnetic field would drastically perturb the anisotropy barrier. For both compositions the highest *T_B_* values are observed for embedded ferrite particles in amorphous silica, and the lowest value corresponds to those particles encased in the MCM-type matrix. Particles in the MCM matrix present the highest *H_K_* value; therefore, their anisotropy barrier is less affected by the presence of an external field than in the other cases. Also, their low anisotropy barrier would account for the low *T_B_* value. Although the anisotropy constant presents the highest value for NiMH and ZnMH samples, their effective magnetic volume may be very small, probably due to three reasons: the small particle size, the presence of an important surface layer of disordered spins, and almost minimized dipolar interactions. The narrow maximum of the ZFC curves and the upturn of the FC curve at low temperatures (observed for ferrite particles encased in MCM-type matrix) clearly reveal an absence of important dipolar interactions in these samples [42]. On the contrary, particles embedded in amorphous silica present the highest *T_B_* value, although this difference is less pronounced in the case of zinc ferrite composition. In the case of nickel ferrite samples, the extremely high *T_B_* value for NiM5 (in comparison with that corresponding to the Ni3 sample) may be understood, considering its probable higher anisotropy barrier *KV* (coming from a slightly higher *K* value, and also probably *V* value as a consequence of its higher particle size). In addition, embedded nanoparticles present a higher *H_K_* value, so their anisotropy barrier would be less perturbed in the presence of an external field.

On the other hand, dipolar interactions (resulting in a broad maximum of the ZFC curves) may be the main factor responsible for the high magnetization values exhibited by bare particles (see Figure 4). Furthermore, the high magnetic response observed at 300 K for Ni3 in contrast to the Zn4 sample (see the susceptibility values in Figure 4), as a result of intense dipolar interactions in the former case, it should be emphasized. The intense dipolar interactions among particles’ moments in the Ni3 sample do not allow the thermal energy to destroy the magnetic ordering among them, even at 300 K.

**Figure 5:**
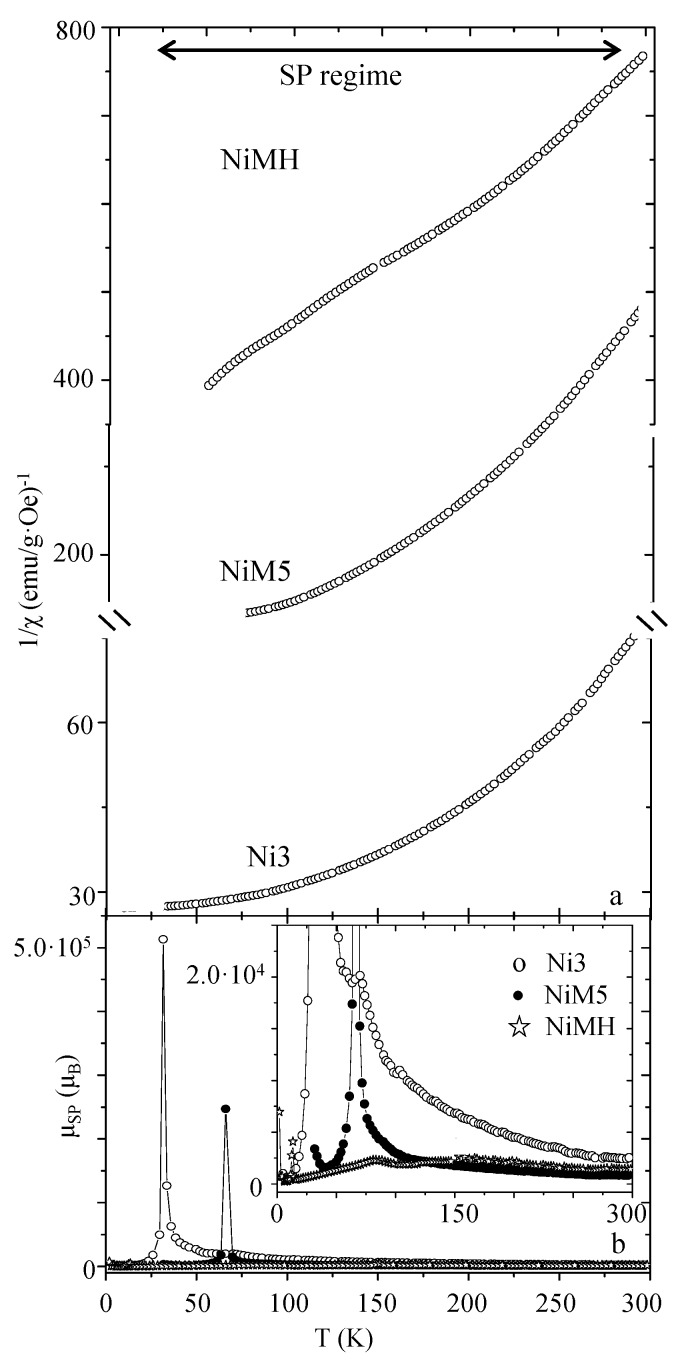
Inverse of susceptibility of nickel ferrite samples (**a**) and superparamagnetic moment dependence with the temperature (**b**); a magnification of the graph is shown in the inset (“SP” means “superparamagnetic”).

**Figure 6:**
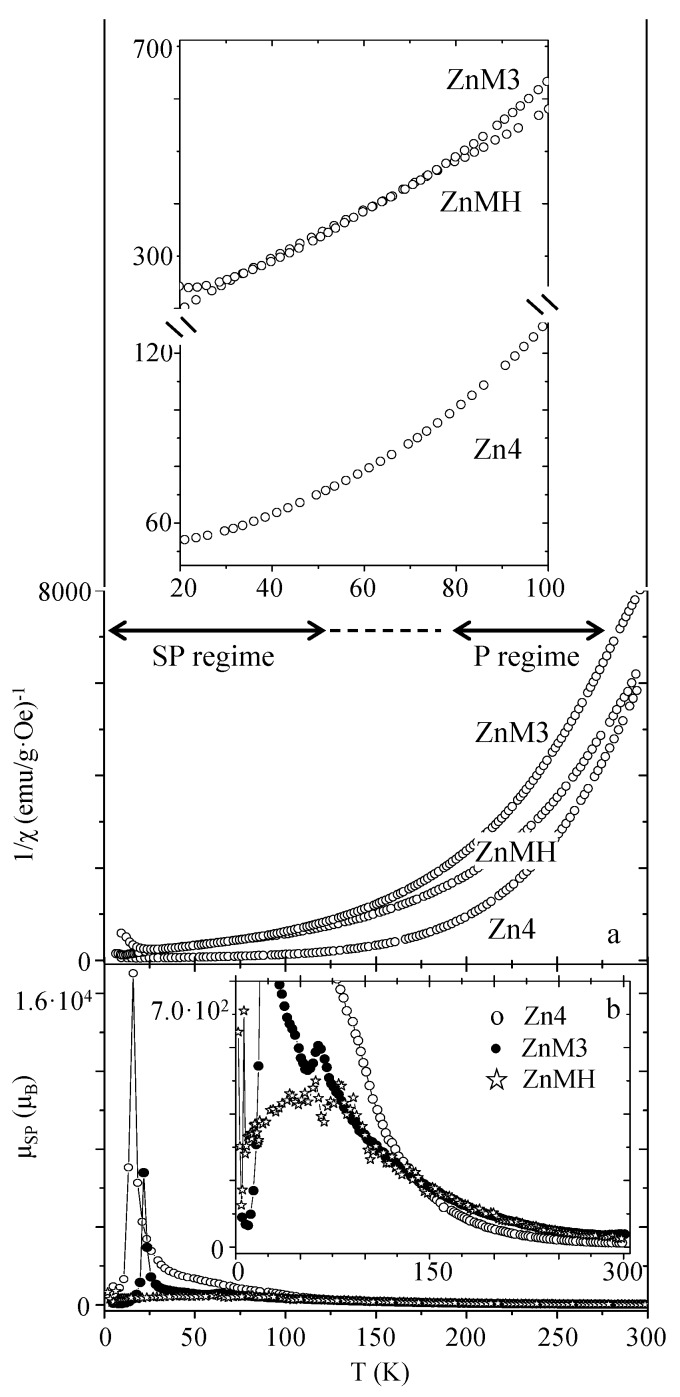
Inverse of susceptibility of zinc ferrite samples (**a**). Insets of (**a**) illustrate a magnification of the graphs. Superparamagnetic moment dependence with the temperature is shown in (**b**). “SP” and “P” mean “superparamagnetic” and “paramagnetic”, respectively.

The inverse of susceptibility has been depicted for nickel ferrite and zinc ferrite samples (Figure 5 and Figure 6, respectively).

This kind of plot provides a useful tool for evaluating the superparamagnetic (SP) to paramagnetic (P) transition, as well as the intensity of dipolar interactions responsible for the different superparamagnetic moments (μ_SP_). This parameter can be calculated from the following expression [43]:

(5) $$\mu_{SP}(\mu_{B})(T)=\frac{3\kappa_{B}}{M_{S}}{\left[\frac{d(1/\chi )}{dT}\right]}^{-1}$$

which indicates that the superparamagnetic moment is dependent on the temperature [19]. If the inverse of the susceptibility illustrates a linear tendency, *μ_SP_* would be constant with the temperature. As can be observed, while 1/*χ* vs. *T* illustrates an almost linear tendency in the case of the NiMH sample, for NiM5 and Ni3 it draws a curve (see Figure 5a). This clearly indicates an almost constant effective magnetic moment in the superparamagnetic temperature range for the NiMH sample, as well as a superparamagnetic moment dependent on the temperature for the Ni3 and NiM5 samples (as can be observed in the *μ_SP_*(*T*) graphs depicted in Figure 5b). The role of the MCM-type matrix, consisting of minimizing the dipolar interactions of encased particles, would be responsible for this observation. The experimental curves are a direct consequence of the temperature effect, especially for the Ni3 and NiM5 samples. When the temperature increases from 5 K, the thermal energy favors particle moment rotation alignment with the applied magnetic field, while an increase of the *μ_SP_* is observed up to a maximum close to the *T_B_* that corresponds to the temperature for which the ordering among the particles is optimal. When the temperature further increases, the thermal energy has an opposite effect and starts to destroy the order among the particles, thus minimizing the *μ_SP_* value. The extremely high *μ_SP_* values for the Ni3 and NiM5 samples account for the large number of interacting particles that contribute to the total magnetization (in comparison with NiMH). In addition, after comparison of the *μ_SP_*, it is worth emphasizing that the MCM silica porous structure would be the matrix that better minimizes these kinds of interactions, probably due to a proper distribution of the particles along the channels. Also, when no matrix is employed to disperse the particles, the decrease of *μ_SP_*due to thermal energy action is slowed down with the temperature. That is, dipolar interactions can be observed in a broader temperature range in the case of bare particles.

A very different conclusion can be drawn when evaluating the 1/*χ* (*T*) graphs for the zinc ferrite samples (shown in Figure 6a). In contrast with the NiFe_2_O_4_ samples, the 1/*χ* (*T*) curves are quite similar for the three studied samples (see Figure 6a). Experimental data of the ZnFe_2_O_4_ particles in the 250 to 300 K temperature range can be fitted to the Curie-Weiss law, obtaining an effective magnetic moment value close to 5.91 *μ_B_* for the three samples, which corresponds to the effective paramagnetic moment characteristic of Fe^3+^ (d^5^) calculated from $\mu =\sqrt{S\left(S+1)\right)}$ (considering the spin only contribution) [22]. Therefore, for this temperature range (250–300 K), NiFe_2_O_4_ samples behave as superparamagnetic, while ZnFe_2_O_4_ particles present an absence of internal magnetic ordering. The superparamagnetic moment dependence with the temperature has been calculated from Equation (5) for the Zn4, ZnM3, and ZnMH samples, and it has been depicted in Figure 6b. In the case of the Zn4 sample, higher *μ_SP_* values can be observed in accordance with greater interaction between dipoles in the sample. As already observed in the NiFe_2_O_4_ samples, the optimal minimization of the dipolar interactions is achieved by the employment of the porous MCM-type matrix, and the highest *μ_SP_* magnitude is found for a temperature value close to the blocking one (for which dipolar interactions are optimal). Also, taking into account that both ferrite systems present similar *M_S_* values, the lower effective *μ_SP_* value observed in the ZnFe_2_O_4_ particles (in comparison with those of nickel ferrite) may be due to low dipolar interactions, characteristic of the zinc ferrite system.

## 3. Experimental Section

### 3.1. Synthesis of the MCM-41 Type Mesoporous Matrix

Mesoporous silica MCM-41 was synthesized by the hydrothermal method [44] (using tetraethylorthosilicate (TEOS) and hexadecyltrimethylammonium bromide (CTAB) as a silica source and template, respectively), in a TEOS:CTAB:H_2_O molar ratio of 1:0.15:120. Stoichiometric amounts of CTAB and H_2_O are mixed and ethylamine (EA) was added afterwards to reach pH 11. Then, TEOS was added dropwise and the mixture placed under stirring for 4 h at room temperature. The resulting gel was heated at 80 °C for 30 min, and was afterwards transferred into an autoclave to be hydrothermally treated at 100 °C for 24–48 h. The obtained white product was filtered, washed with deionized water, and dried in air. Finally, the material was treated at 500 °C in air for 6 h, with a heating rate of 1 °C/min.

### 3.2. Preparation of Ferrite Nanoparticles Encapsulated in the Silica Mesoporous Matrix

Two 0.165 M aqueous solutions containing M(II) nitrate (M:Ni, Zn) were prepared and mixed with a 0.165 M aqueous solution containing Fe(III) nitrate in a M:Fe molar ratio of 1:2. The final solution was infiltrated in approximately 1.0 g of the MCM-41 type material with a final volume of 5 mL. The infiltrated materials were kept at room temperature for 24 h and treated afterwards at 600 °C for 2 h. After this treatment, ferrite nanoparticles inside the porous network of the matrix were obtained. These encased NiFe_2_O_4_ and ZnFe_2_O_4_ nanoparticles in the MCM-41 type matrix were labeled as NiMH and ZnMH samples, respectively.

NiFe_2_O_4_ and ZnFe_2_O_4_ nanoparticles considered for this study have been already prepared through the solvothermal method [18,22] and embedded particles in amorphous silica [10,30]. They have been labeled as Ni3 and Zn4 (bare particles) and NiM5 and ZnM3 (embedded particles), where Ni and Zn refers to the M^2+^ cation of MFe_2_O_4_ composition and the number refers to the mean particle size estimated from TEM images. Their full characterization can be found in [18,22] (samples Ni3 and Zn4, respectively), as well as [10,30] (samples NiM5 and ZnM3, respectively).

### 3.3. Preparation of Ferrite Nanoparticles through Solvothermal Conditions

Already prepared NiFe_2_O_4_ and ZnFe_2_O_4_ nanoparticles have been also considered for this study. They were prepared through the solvothermal method following the details contained in [18,22], respectively. Stoichiometric amounts of the corresponding nitrates were dissolved in ethylene glycol or glycerol in a 10^−5^ M and 10^−4^ M concentration for NiFe_2_O_4_ and ZnFe_2_O_4_, respectively. Afterwards, the hydroxides were precipitated with 0.5 M and 2.0 M KOH for NiFe_2_O_4_ and ZnFe_2_O_4_, respectively, and the obtained brown mixture was transferred into a stainless steel autoclave to be solvothermally treated at 180 °C and 160 °C for 3 h and 168 h (for NiFe_2_O_4_ and ZnFe_2_O_4_, respectively). The obtained product was recovered after filtering and washing with distilled water and ethanol. The samples were named as Ni3 and Zn4, in reference to the M^2+^ ferrite cation and particle size in nm.

### 3.4. Preparation of Ferrite Nanoparticles Embedded in Amorphous Silica

Embedded NiFe_2_O_4_ and ZnFe_2_O_4_ particles in amorphous silica (in a 30/70 ferrite/silica weight ratio) were also considered for this study and were synthesized as [10,30] indicate, respectively. Stoichiometric amounts of metal nitrates were dissolved in ethanol, and later distilled water and tetraethylorthosilicate (TEOS) were added in a molar ratio of TEOS/ EtOH/H_2_O of 1:4:11.67.24 during a gelling period of 4 days; the precursor of SiO_2_ (TEOS) polymerizes to give a solid silica network, in which the metal nitrates are distributed. Different portions of the gel were subjected to thermal treatments in air atmosphere at 700 °C for 12 h and 600 °C for 12 h for NiFe_2_O_4_ and ZnFe_2_O_4_, respectively. They have been labeled as NiM5 and ZnM3, in reference to the M^2+^ ferrite cation (amorphous silica as matrix and particle size in nm).

### 3.5. Characterization Techniques

The structural characterization was carried out by X-ray diffraction. To characterize the silica matrices (20 mg sample mass, approximately), low-angle diffractograms were recorded using a *Panalytical X’Pert PRO MPD* (40 mA, 45 kV) powder diffractometer with Cu Kα radiation. The ferrite nanoparticles encapsulated in the silica mesoporous matrices were characterized using a Siemens D-5000 powder diffractometer (25 mA, 35 kV) with Cu Kα radiation (20 mg sample mass, approximately). Morphological characterization was made by Transmission Electron Microscopy (TEM), employing a JEOL-JEM-2100 microscope working at 200 kV and equipped with a EDX (Energy Dispersive X-ray Spectroscopy) detector for compositional analysis. Textural characterization was done by gas adsorption with a surface area analyzer ASAP 2020 (Micromeritic Instrument Corporation, Norcross, GA, USA). Prior to the measurements, the samples were outgassed at 110 °C and ≈1 × 10^−3^ Torr for 3 h. Magnetic measurements of powder samples were made in a XL-SQUID magnetometer (Quantum Design, San Diego, CA, USA), taking into account the ferrite:matrix weight ratio deduced from EDX measurements. The zero field cooling (ZFC) and field cooling (FC) magnetic susceptibility was measured in the temperature range of 2–300 K and the magnetic field of 500 Oe. The magnetization curves were recorded at 5 K and 250 K and magnetic field up to 5 T.

## 4. Conclusions

NiFe_2_O_4_ and ZnFe_2_O_4_ particles (of 2.6 nm approximately) have been prepared, encased in the MCM-type matrix by a wet impregnation of the reactants. Their magnetic behavior has been analyzed and compared with that corresponding to particles of the same composition and a similar size. For such analyses the ferrite:matrix weight ratio (deduced from EDX measurements) has been taken into account in all the cases.

The role of the MCM matrix has been deeply investigated. It can be asserted that it drastically modifies the magnetic properties of the encased particles as they behave magnetically harder. Thus, particles contained in the porous structure illustrate lower magnetization values (as consequence, among others, of an important non-magnetic layer, higher anisotropy constant resulting from the increment of the surface anisotropy contribution, and higher anisotropy fields) for both compositions. The study also indicates a better minimization of dipolar interactions in ferrite particles when employing the porous MCM structure rather than the amorphous silica, since in the latter case particles exhibit larger superparamagnetic moments.

## Figures and Tables

**Table 1:** Magnetic parameters corresponding to the different prepared samples.

Sample		*M_S_* (emu/g)	*K* (erg/cm^3^)	*H_K_* (Oe)	*H_C_*_,5K_ (Oe)	*T_B_* (K)
Ni3	3 nm bare particles	46.8	1.7 × 10^5^	1312	286	27
NiMH	2.6 nm particles in the MCM-type matrix	16.0	7.1 × 10^5^	16492	880	13
NiM5	5 nm particles in amorphous SiO_2_	20.9	2.3 × 10^5^	3912	1390	63
Zn4	4 nm bare particles	43.1	2.5 × 10^5^	1722	125	14
ZnMH	2.6 nm particles in the MCM-type matrix	22.0	9.1 × 10^5^	15516	400	6.5
ZnM3	3 nm particles in amorphous SiO_2_	15.9	2.3 × 10^5^	5311	1300	23

*M_S_* = saturation magnetization; *K* = anisotropy constant; *H_K_* = anisotropy field; *H_C_*_,5K_ = coercive field at 5 K; *T_B_* = blocking temperature for an applied magnetic field of 500 Oe.

## Supplementary Materials

The following are available online at http://www.mdpi.com/2079-4991/7/7/151/s1, Figure S1: Adsorption-desorption isotherms, cumulative pore volume and pore size distribution of the samples, Table S1: Textural parameters for the prepared MCM-type matrix and the composites.
