# Supplementary material for: Mesoporous Silica Matrix as a Tool for Minimizing Dipolar Interactions in NiFe2O4 and ZnFe2O4 Nanoparticles
Source: Nanomaterials (Basel). 2017 Jun 22;7(7):151. doi: 10.3390/nano7070151 (PMC5535217; doi:10.3390/nano7070151)
Supplement: Supplementary file 1 [file nanomaterials-07-00151-s001.pdf]

The MCM matrix as a reducing agent of dipolar interactions in $NiFe_2O_4$ and $ZnFe_2O_4$ nanoparticles.

M.Virumbrales, R. Saez-Puche, M. J. Torralvo, V. Blanco-Gutierrez

Departamento de Química Inorgánica, Facultad de Ciencias Químicas, Universidad Complutense de Madrid, 28040 Madrid, Spain

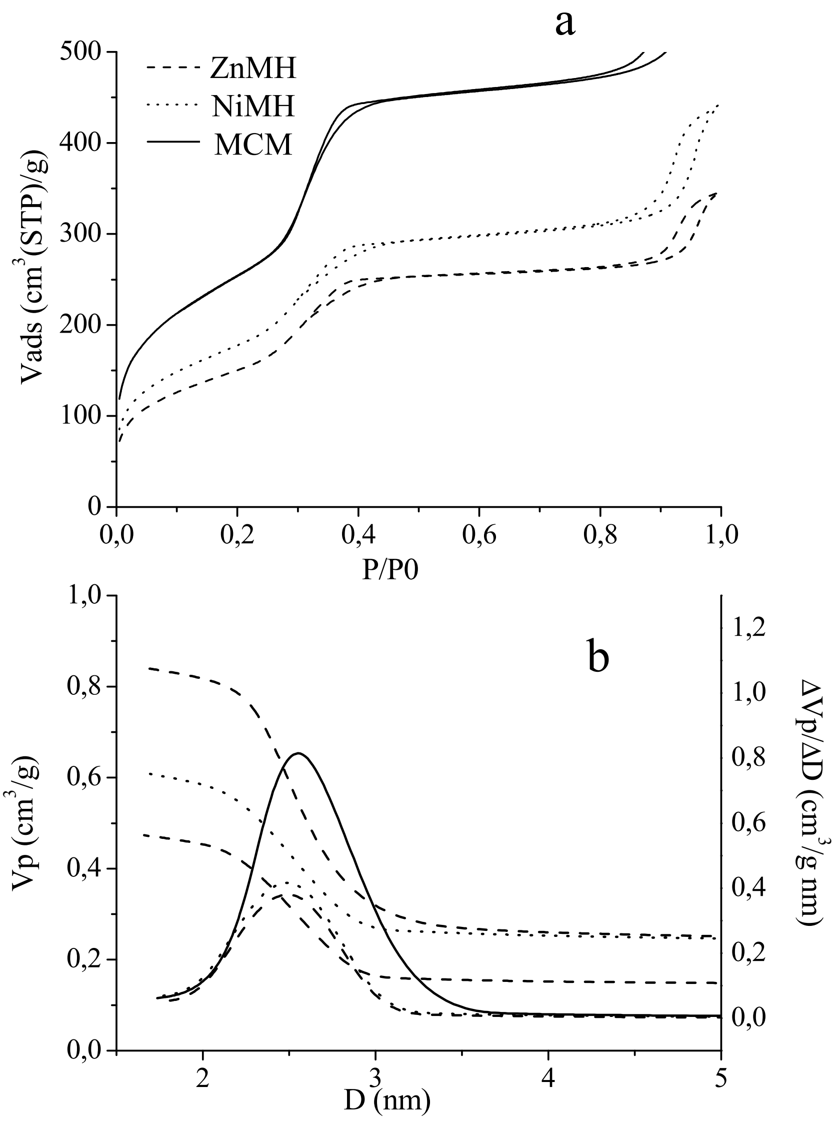

Figure S1. Adsorption-desorption isotherms (a) and cumulative pore volume and pore size distribution (b) of the samples.

Table S1. Textural parameters for the prepared MCM-type matrix and the composites.

Sample	S_{BET} (m^2/g)	V_p (cm^3/g)	d_{pore} (nm)
MCM	925	0.84	2.6
NiMH	648	0.61	2.5
ZnMH	547	0.47	2.5
